# Seroprevalence of SARS-CoV-2 antibodies in schoolchildren in the city of São Paulo, 2020

**DOI:** 10.11606/s1518-8787.2023057004782

**Published:** 2023-05-11

**Authors:** Gabriela Akemi Kamioka, Geraldine Madalosso, José Olimpio Moura de Albuquerque, Selma Anequini Costa, Paula Bisordi Ferreira, Ana Paula Sayuri Sato, Paula Regina Glasser, Francisco Alberto Pino, Patrícia Carla Piragibe Ramos Burihan, Ana Carolina Aguiar de Carvalho, Ana Beatriz Pagliaro Amorim, Cinthya Luzia Cavazzana, Caroline Cotrim Aires, Ana Paula Arruda Geraldes Kataoka, Elisa San Martin Mouriz Savani, Thirsa Alvares Franco Bessa, Breno Souza de Aguiar, Marcelo Antunes Failla, Edson Aparecido dos Santos, Edjane Maria Torreão Brito, Maria Cristina Honório dos Santos, Luiz Artur Vieira Caldeira, Solange Maria Saboia e Silva, Luiz Carlos Zamarco, Sandra Maria Sabino Fonseca, Marcia Maria de Cerqueira Lima, Ivanilda Argenau Marques, Athenê Maria de Marco França Mauro, Eduardo de Masi

**Affiliations:** I Secretaria Municipal de Saúde Coordenadoria de Vigilância em Saúde São Paulo SP Brasil Secretaria Municipal de Saúde. Coordenadoria de Vigilância em Saúde. São Paulo, SP, Brasil; II Universidade de São Paulo Faculdade de Saúde Pública São Paulo SP Brasil Universidade de São Paulo. Faculdade de Saúde Pública. São Paulo, SP, Brasil; III Secretaria de Agricultura e Abastecimento Instituto de Economia Agrícola São Paulo SP Brasil Secretaria de Agricultura e Abastecimento. Instituto de Economia Agrícola. São Paulo, SP, Brasil; IV Secretaria Municipal de Saúde São Paulo SP Brasil Secretaria Municipal de Saúde. São Paulo, SP, Brasil

**Keywords:** Child, Adolescent, COVID-19, epidemiology, SARS-CoV-2, Seroepidemiologic Studies

## Abstract

**OBJECTIVE:**

To estimate seroprevalence of SARS-CoV-2 antibodies in schoolchildren aged 4 to 14 years living in the city of São Paulo, according to clinical, demographic, epidemiological, and social variables, during the school closure period as a measure against covid-19 spread.

**METHODS:**

A serological survey was made in September 2020 with a random sample stratified by school system (municipal public, state public and private) type. A venous blood sample was collected using the Wondfo SARS-CoV-2 Antibody Test (lateral flow method) for detection of total SARS-CoV-2 virus antibodies. Semi-structured questionnaires were applied to collect clinical, demographic, social, and epidemiological data.

**RESULTS:**

Seroprevalence of SARS-CoV-2 antibodies in schoolchildren was of 16.6% (95%CI 15.4–17.8). The study found higher seroprevalence in the municipal (18.5%; 95%CI 16.6–20.6) and state (16.2%; 95%CI 14.4–18.2) public school systems compared to the private school system (11.7; 95%CI 10.0–13.7), among black and brown students (18.4%; 95%CI 16.8–20.2) and in the most vulnerable social stratum (18.5 %;95%CI 16.9–20.2). Lower seroprevalence was identified in schoolchildren who reported following the recommended protective measures against covid-19.

**CONCLUSION:**

Seroprevalence of SARS-CoV-2 antibodies is found mainly in the most socially vulnerable schoolchildren. This study can contribute to support public policies that reinforce the importance of suspending face-to-face classes and developing strategies aimed at protective measures and monitoring of the serological status of those who have not yet been included in the vaccination schedule.

## INTRODUCTION

The World Health Organization (WHO) has declared covid-19 – a disease caused by the SARS-CoV-2 virus – a pandemic in March 2020^1^. The epidemiology, disease clinical characterization, care, and evolution of covid-19 cases are considered challenges to public health worldwide, therefore areas of study that are in great demand. The disease affected all age groups in different proportions and degrees of clinical manifestations^2^. Studies show that children have milder symptoms and a more favorable prognosis when compared to other age groups^3^.

In this sense, age has been an important factor associated with the disease severity and mortality^4^. In the first months of the pandemic, only 2.1% covid-19 cases were reported in children under 18 years of age, and no deaths were reported in children under 9 years of age. Among the notified cases, approximately 4.4% had the severe form of the disease^5^ and about 1.0% people were hospitalized or died. Studies have shown up to 14.6% seroprevalence of SARS-CoV-2 antibodies in European countries’ children and 75.2% in the United States^6,7^. Differences in the distribution, maturation, and functionality of angiotensin-converting enzyme 2 (ACE2) receptors and high levels of lymphocytes in the children’s blood, especially natural killer cells, may be greater when compared to adults^8^.

The suspension of face-to-face classes, with the consequent school closure between 2020 and 2021, was a measure adopted in almost all parts of the world to prevent and contain covid-19 spread^9^. It caused a significant social and economic impact on society at regional, national, and global levels^10^. In Brazil, actions were implemented in March 2020 towards social distancing, and there was replacement of face-to-face classes with distance learning, voluntary quarantine, and interruption of non-essential services.

In May 2020, when there was a gradual school and teaching unit reopening in the city of São Paulo, knowing the seroprevalence of SARS-CoV-2 antibodies in children and adolescents was extremely important to making safe decisions about reopening services and teaching units and mitigating the disease transmission^11^.

In June 2020, the Municipal Health Secretariat of São Paulo started making the serological survey for SARS-CoV-2 in adults^12^, and, maintaining the epidemiological surveillance actions, the serological survey was made in schoolchildren in September. This study used data from the schoolchildren survey and aimed to estimate the seroprevalence of total SARS-CoV-2 antibodies in schoolchildren aged 4 to 14 years old, residing in the city of São Paulo in 2020, according to clinical, demographic, epidemiological, and social variables.

## METHODS

It is a serological survey in schoolchildren between 4 and 14 years of age, living in the city of São Paulo.

### Study Area

São Paulo is the most populous city in Brazil and 2019 had an estimated population of 11.8 million inhabitants, with 1.6 million aged between 4 and 14 years old. The city presents great economic and social disparity, reflecting heterogeneity in education, income, and housing conditions. The human development index (HDI) of the areas covered by the 472 basic health centers ranges from 0.62 (with 147 areas of centers in the HDI range of 0.62 to 0.73) to 0.95 (with 83 areas of centers in the HDI range of 0.83 to 0.95). The city, capital of the state of São Paulo, is divided into six health regions, which also have a wide variation in HDI. The areas of health centers with the lowest HDI values are concentrated in the South, East, and North regions, located at the city extremes, while the highest values are centralized in the Southeast, Center and West regions, areas closer to downtown. Therefore, social inequality in the city follows a concentric pattern, with the most vulnerable population tending towards the outskirts and the wealthiest population towards downtown, in the direction of the West region^13^.

### Study population

Sample participants were students registered on the database of public (municipal and state) and private school systems of the Municipal and State Education Secretariats of São Paulo, considering the following inclusion criteria: children aged 4 to 14 years old, living in the city of São Paulo.

### Sampling Design

Random sampling^14^ was drawn, stratified according to the school system, each system as a sampling domain (municipal public school system, state public school system, and private school system).

For municipal and state systems, the minimum sample size was planned to obtain 16% prevalence estimates, with a variation coefficient of less than 15%, at a 95% confidence level. For the private system, the sample was calculated to obtain a 9% prevalence estimate, also with a variation coefficient of less than 15% and a 95% confidence level. In this way, a minimum sample size of 900 students was established for each of the public systems, and 1,200 students for the private system. For the student selection, there was compensation for the expected 50% nonresponse rate.

### Testing and Interview

Data collection was carried out between September 1 and 16, 2020, at the selected child’s home, since there were no face-to-face classes when the study was conducted. The interview was carried out with the child and a guardian present at the time of the visit, using a semi-structured data collection form, which included questions about socioeconomic, demographic, epidemiological, and disease prevention measures information. The collected data were included in electronic form on the FormSUS/DATASUS platform, version 3.0, by the health center in charge.

To collect a sample of venous blood (serum), Wondfo SARS-CoV-2 Antibody Test^®^ (lateral flow method) (Guangzhou Wondfo Biotech Co., Ltd.) was used, which detects total SARS-CoV-2 antibodies (IgG/IgM) in human whole blood, serum and plasma, with 86.4% sensitivity and 99.6% specificity. In Brazil, the Ministry of Health made the same test available under the name One Step Covid-2019 Test^®^, through the manufacturer Celer Biotecnologia S/A’s legal representative^15^.

### Data Analysis

Seroprevalence of SARS-CoV-2 antibodies weighted by the total collections performed by stratum and the respective 95% confidence intervals (95%CI) was estimated according to the sampling design using statistical packages, namely, R and Stata version 13.

Seroprevalence estimates and respective 95%CI were obtained for each sample stratum and according to health region; sex; race/color; guardians’ schooling; social stratification; number of residents in the housing unit; student age group; presence or absence of the following symptoms at the time of the interview: loss of smell, loss of taste, fever, weakness, cough, headache, runny nose, nausea, diarrhea, dyspnea, sore throat, and shortness of breath; measures taken during the pandemic; place where they stayed most of the time – at home or with a family member and/or neighbor; coexistence or non-coexistence with people over 60 years of age in the same housing unit; adoption or non-practice of social distancing; existence or non-existence of a resident in the housing unit who worked outside the home; contact or lack of contact with a suspected or confirmed covid-19 case; use of public transportation, and use of a face mask.

Social stratum was classified according to the family income informed by the interviewee. Upper class was defined as that in which income was greater than R$ 8,640.00; middle class was that with income between R$ 2,005.00 and R$ 8,640.00; and lower class was the one in which the income was less than R$ 2,005.00.

To compare the frequencies between the categories of each variable, Rao-Scott Chi-Square test was used, considering a 5% significance level.

Quantum Geographic Information System (QGIS) software version 3.22 was used to analyze the spatial distribution of the seroprevalence of SARS-CoV-2antibodies in schoolchildren in the city of São Paulo.

### Ethical Aspects

The study was approved by the National Research Ethics Committee (CAAE 36032820.3.0000.0008). Those responsible for the students selected to participate in the study were duly informed about the objectives, risks, benefits, and the right to refuse. The collection of blood samples and the interview were only carried out after the informed consent form was signed by the guardian and the assent term by the student under 18 years of age.

## RESULTS

The study included 4,198 schoolchildren, aged between 4 and 14 years old, residing in the city of São Paulo, 1,469 from the municipal school system, 1,521 from the state school system, and 1,208 from the private school system.

The seroprevalence of total SARS-CoV-2 antibodies was of 16.6% (95%CI 15.4–17.8), with 18.5% (95%CI 16.6–20.6) in the municipal system, 16.2% (95%CI 14.4–18.2) in the state system, and 11.7% (95%CI 10.0–13.7) in the private system. There was a statistical difference between seroprevalence in school systems (p<0.001).

According to the city region, seroprevalence estimates were different only for the private system (p = 0.003), with lower seroprevalence in the Southeast region (5.8%; 95%CI 3.4–9.5), and higher in the South region (16.9%; 95%CI 13.1–21.4) (Table 1).

**Table 1:** Distribution and seroprevalence of SARS-CoV-2 antibodies in schoolchildren according to demographic factors and school system. City of São Paulo, 2020.

Demographic variables	TOTAL		Municipal public school system		State public school system		Private school system	
			
	n (%)	Seroprevalence (95%CI)	n (%)	Seroprevalence (95%CI)	n (%)	Seroprevalence (95%CI)	n (%)	Seroprevalence (95%CI)
Total	4,198 (100.0)	16.6 (15.4–17.8)^a^	1,469 (45.7)	18.5 (16.6–20.6)	1,521 (38.2)	16.2 (14.4–18.2)	1,208 (16.1)	11.7 (10.0–13.7)
Region								
Central-West	294 (6.3)	18.1 (13.7–23.5)^a^	92 (6.3)	22.2 (14.8–32.0)	67 (4.4)	18.2 (10.6–29.3)	135 (11.2)	11.5 (7.0–18.1)^a^
North	848 (20.1)	18.2 (15.6–21.1)	307 (20.9)	18.9 (14.8–23.7)	281 (18.5)	20.6 (16.2–25.7)	259 (21.4)	11.4 (8.1–15.9)
Southeast	727 (16.6)	12.4 (10.0–15.2)	213 (14.5)	13.8 (9.8–19.2)	264 (17.4)	14.2 (10.4–19.1)	250 (20.7)	5.8 (3.4–9.5)
East	1,094 (27.3)	16.6 (14.4–19.1)	446 (30.4)	19.6 (16.2–23.6)	405 (26.6)	14.1 (11.1–17.9)	243 (20.1)	11.6 (8.1–16.3)
South	1,235 (29.7)	17.4 (15.3–19.7)	410 (27.9)	18.7 (15.1–22.8)	504 (33.1)	16.3 (13.3–19.8)	321 (26.6)	16.9 (13.1–21.4)
Sex								
Male	2,071 (49.7)	17.9 (16.2–19.7)	743 (50.6)	19.9 (17.2–23.0)	739 (48.8)	17.3 (14.7–20.2)	589 (49.1)	13.3 (10.8–16.3)
Female	2,113 (50.3)	15.3 (13.7–17.0)	724 (49.4)	17.1 (14.5–20.0)	777 (51.2)	15.2 (12.8–17.9)	611 (50.9)	10.4 (8.2–13.1)
No information	14 (–)	–	1 (–)	–	5 (–)	–	8 (–)	–
Age group								
4 and 5 years	954 (21.8)	16.8 (14.4–19.5)	393 (27.4)	18.0 (14.5–22.1)	139 (9.3)	16.7 (11.3–23.8)	421 (35.5)	14.3 (11.3–18.0)
6 to 10 years	1,875 (45.6)	16.5 (14.8–18.4)	561 (39.2)	20.3 (17.2–23.9)	817 (54.7)	15.2 (12.9–17.9)	497 (41.9)	10.3 (7.9–13.3)
11 to 14 years	1,284 (32.6)	15.9 (13.9–18.1)	478 (33.4)	16.3 (13.2–19.9)	538 (36.0)	17.0 (14.0–20.4)	268 (22.6)	10.2 (7.1–14.4)
No information	85 (–)	–	36 (–)	–	27 (–)	–	22 (–)	–
Race/color								
White	2,046 (46.9)	14.5 (12.9–16.3)^a^	654 (44.8)	16.9 (14.2–20.0)	648 (42.9)	15.2 (12.6–18.2)	431 (36.2)	8.5 (6.7–10.8)^a^
Black/brown	2,082 (52.4)	18.4 (16.8–20.2)	796 (54.5)	19.7 (17.1–22.7)	854 (56.6)	17.1 (14.7–19.8)	744 (62.4)	17.6 (14.3–21.6)
Others	35 (0.7)	18.2 (7.6–37.3)	10 (0.7)	30.0 (10.0–62.4)	8 (0.5)	12.5 (1.7–53.7)	17 (1.4)	6.3 (0.9–33.5)
No information	35 (–)	–	8 (–)	–	11 (–)	–	16 (–)	–
Number of residents in the housing unit								
1 to 2	175 (4.5)	16.8 (14.4–19.5)	58 (4.3)	15.8 (8.4–27.7)	60 (4.3)	10.2 (4.6–20.8)	57 (5.3)	12.7 (6.2–24.4)
3 to 4	2,333 (59.3)	16.5 (14.8–18.4)	745 (56.0)	17.7 (15.1–20.6)	813 (58.3)	17.0 (14.5–19.7)	774 (71.3)	12.0 (9.9–14.5)
5 +	1,303 (36.2)	15.9 (13.9–18.1)	528 (39.7)	21.1 (17.8–24.8)	521 (37.4)	15.4 (12.5–18.8)	254 (23.4)	11.3 (7.9–15.9)
No information	387 (–)	–	137 (–)	–	127 (–)	–	123 (–)	–
Social stratification								
Upper class	156 (2.4)	5.2 (2.4–11.0)^a^	12 (0.8)	8.3 (1.2–41.3)	6 (0.4)	16.7 (2.3–63.1)^a^	138 (11.6)	3.6 (1.5–8.4)^a^
Middle class	1,379 (30.9)	12.6 (10.8–14.6)	403 (27.7)	15.4 (12.1–19.3)	429 (28.4)	11.1 (8.4–14.4)	388 (32.5)	10.1 (7.8–12.9)
Lower class	2,275 (58.6)	18.5 (16.9–20.2)	924 (63.4)	19.6 (17.2–22.3)	963 (63.9)	17.3 (15.0–19.9)	546 (45.8)	17.7 (14.2–21.9)
Uniformed	348 (8.1)	19.2 (15.1–24.1)	118 (8.1)	20.2 (13.8–28.5)	110 (7.3)	22.4 (15.5–31.3)	120 (10.1)	11.3 (6.7–18.5)
No information	40 (–)	–	11 (–)	–	13 (–)	–	16 (–)	–
Most educated resident’s schooling								
No schooling	15 (0.4)	34.5 (14.8–61.4)^a^	3 (0.2)	33.3 (4.3–84.7)	8 (0.6)	37.5 (12.5–71.5)^a^	4 (0.3)	25.0 (3.4–76.2)^a^
Primary education	345 (9.3)	23.1 (18.9–28.1)	138 (9.9)	21.7 (15.6–29.4)	156 (10.9)	24.5 (18.3–32.0)	51 (4.4)	24.5 (14.5–38.3)
High school	2,266 (60.0)	18.1 (16.5–19.8)	904 (64.7)	19.7 (17.2–22.4)	916 (63.7)	16.7 (14.4–19.3)	665 (57.0)	15.8 (12.7–19.6)
Higher education	1.375 (30.3)	11.3 (9.6–13.3)	352 (25.2)	14.8 (11.4–18.9)	357 (24.8)	10.3 (7.5–13.9)	446 (38.3)	8.1 (6.3–10.5)
No information	197 (–)	–	71 (–)	–	84 (–)	–	42 (–)	–

^a^ p-value < 0.05.

**Figure 1:**
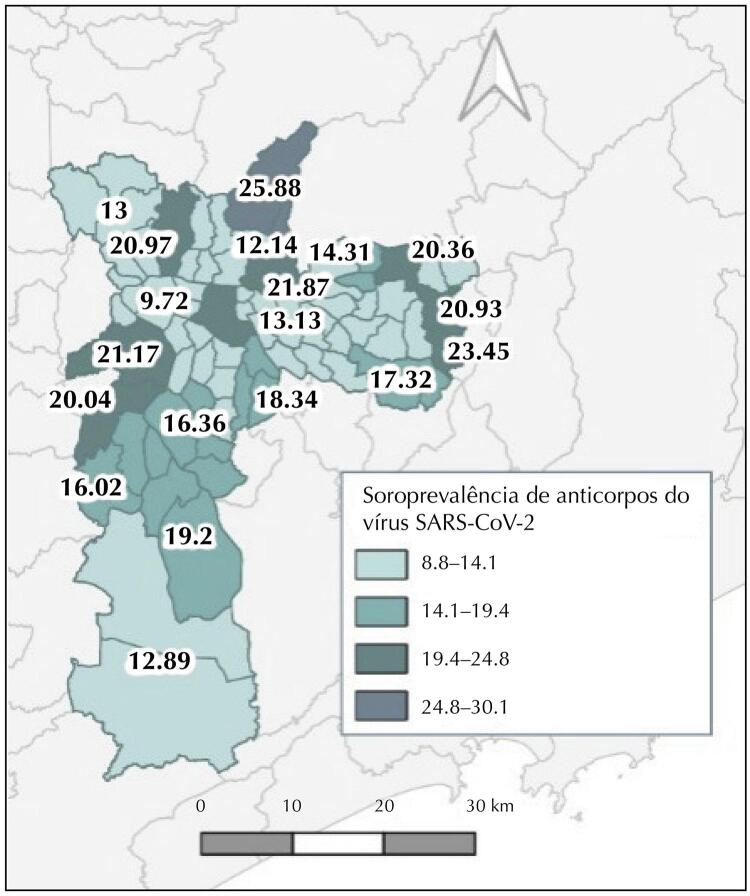
Spatial distribution of seroprevalence of SARS-CoV-2 antibodies in schoolchildren, according to the Health Surveillance Center. City of São Paulo.

Figure 1 shows higher seroprevalence of SARS-CoV-2 antibodies in schoolchildren in the peripheral city regions.

There was no difference in seroprevalence regarding sex, age group, and total number of residents in the housing unit. Nevertheless, in the private system, the estimates of seroprevalence in the black or brown race/color were significantly higher (17.6%; 95%CI 14.3–21.6; p<0.001). In public state and private systems, seroprevalence was higher among students who lived with less educated residents (24.5%; 95%CI 18.3–32.0; p<0.001 and 24.5%; 95%CI 14.5–38.3; p<0.001, respectively), and among the most vulnerable students (lower class) (17.3%; 95%CI 15.0–19.9 and 17.7%; 95%CI 14.2–21.9, respectively) (Table 1).

Table 2 presents the estimates of seroprevalence of SARS-CoV-2 antibodies according to clinical variables. The proportion of children with symptoms at the time of the interview was of 29.1%. The main symptoms presented were: runny nose (64.1%), cough (59.3%), sore throat (37.6%), headache (33.2%), and fever (29.8%). Symptoms of loss of smell (55.0%; 95%CI 38.2–70.7; p<0.001), loss of taste (47.3%; 95%CI 33.2–61.8; p<0.001), and fever (26.5%; 95%CI 21.7–32.0) were associated with higher seroprevalence of total SARS-CoV-2 antibodies.

**Table 2:** Distribution and seroprevalence of SARS-CoV-2 antibodies in schoolchildren according to clinical factors and school system. City of São Paulo, 2020.

Variable	TOTAL		Municipal public school system		State public school system		Private school system	
			
	n (%)	Seroprevalence (95%CI)	n (%)	Seroprevalence (95%CI)	n (%)	Seroprevalence (95%CI)	n (%)	Seroprevalence (95%CI)
Presence of symptoms at the time of the interview								
Yes	1,214 (29.1)	15.4 (14.0–16.8)^a^	408 (27.9)	20.7 (17.1–25.0)	466 (30.7)	19.1 (15.8–23.0)^a^	340 (28.3)	15.1 (11.6–19.3)^a^
No	2,968 (70.9)	19.2 (17.0–21.7)	1,054 (72.1)	17.6 (15.4–20.0)	1,050 (69.3)	14.7 (12.7–17.0)	863 (71.7)	10.5 (8.6–12.8)
No information	16 (–)		6 (–)	–	5 (–)	–	5 (–)	–
Search for medical care								
Yes	302 (24.8)	21.9 (17.4–27.3)	95 (24.1)	25.3 (17.5–35.0)	107 (23.6)	20.8 (14.1–29.6)	100 (30.3)	17.0 (10.8–25.7)
No	877 (75.2)	17.9 (15.4–20.7)	300 (75.9)	19.2 (15.1–24.1)	347 (76.4)	18.0 (14.3–22.5)	230 (69.7)	13.5 (9.6–18.7)
No information	35 (–)	–	13 (–)	–	12 (–)	–	10 (–)	–
Loss of smell								
Yes	38 (3.9)	55.0 (38.2–70.7)^a^	12 (3.6)	58.3 (30.6–81.6)^a^	17 (4.4)	50.0 (27.2–72.8)^a^	9 (3.3)	62.5 (28.3–87.5)^a^
No	955 (96.1)	18.9 (16.4–21.7)	325 (96.4)	20.5 (16.4–25.3)	369 (95.6)	19.2 (15.4–23.6)	261 (96.7)	13.3 (9.7–18.1)
No information	221 (–)	–	71 (–)	–	80 (–)	–	70 (–)	–
Loss of taste								
Yes	49 (4.9)	47.3 (33.2–61.8)^a^	14 (4.1)	42.8 (20.6–68.5)	22 (5.7)	50.0 (30.2–69.8)^a^	13 (4.7)	50.0 (24.3–75.7)^a^
No	953 (95.1)	18.9 (16.4–21.7)	323 (95.9)	20.9 (16.8–25.8)	367 (94.3)	19.1 (15.3–23.5)	263 (95.3)	12.8 (9.3–17.5)
No information	212 (–)	–	71 (–)	–	77 (–)	–	64 (–)	–
Fever								
Yes	317 (29.8)	26.5 (21.7–32.0)	105 (29.8)	26.9 (19.3–36.3)	120 (29.2)	28.0 (20.6–36.8)^a^	92 (31.7)	22.0 (14.6–31.7)
No	737 (70.2)	17.1 (14.4–20.2)	248 (70.2)	18.7 (14.3–24.1)	291 (70.8)	17.0 (13.1–21.8)	198 (68.3)	12.9 (8.9–18.5)
No information	160 (–)	–	55 (–)	–	55 (–)	–	50 (–)	–
Weakness								
Yes	95 (8.9)	26.5 (18.2–37.0)	25 (7.4)	28.0 (13.9–48.3)	38 (9.6)	27.0 (15.2–43.4)	32 (11.5)	22.6 (11.1–40.5)
No	916 (91.1)	19.6 (17.0–22.5)	312 (92.6)	21.4 (17.1–26.3)	358 (90.4)	20.1 (16.2–24.6)	246 (88.5)	13.3 (9.6–18.3)
No information	203 (–)	–	71 (–)	–	70 (–)	–	62 (–)	–
Cough								
Yes	656 (59.3)	19.0 (16.0–23.8)	220 (59.9)	22.0 (17.0–28.0)	249 (58.0)	17.7 (13.5–23.0)	187 (60.9)	13.8 (9.5–19.7)
No	447 (40.7)	19.7 (16.1–23.8)	147 (40.1)	19.9 (14.1–27.2)	180 (42.0)	21.0 (15.6–27.7)	120 (39.1)	15.3 (9.8–23.0)
No information	111 (–)	–	41 (–)	–	37 (–)	–	33 (–)	–
Headache								
Yes	342 (33.2)	20.6 (16.4–25.5)	117 (33.7)	23.9 (17.0–32.5)	139 (33.7)	18.8 (13.1–26.3)	86 (30.1)	15.3 (9.1–24.6)
No	703 (66.8)	19.1 (16.2–22.3)	230 (66.3)	19.8 (15.1–25.5)	273 (66.3)	20.4 (16.0–25.7)	200 (69.9)	13.4 (9.3–19.0)
No information	169 (–)	–	61 (–)	–	54 (–)	–	54 (–)	–
Runny nose								
Yes	723 (64.1)	17.5 (14.7–20.6)	249 (65.9)	18.6 (14.2–24.0)	257 (60.1)	18.8 (14.5–24.1)	217 (69.3)	11.3 (7.7–16.4)
No	396 (35.9)	21.7 (17.7–26.3)	129 (34.1)	25.0 (18.2–33.3)	171 (39.9)	19.6 (14.3–26.4)	96 (30.7)	18.3 (11.7–27.5)
No information	95 (–)	–	30 (–)	–	38 (–)	–	27 (–)	–
Nausea								
Yes	81 (8.2)	21.3 (13.3–32.4)	30 (8.9)	26.7 (13.9–45.1)	30 (7.6)	17.2 (7.3–35.4)	21 (7.7)	14.3 (4.6–36.3)
No	925 (91.8)	19.8 (17.2–22.7)	309 (91.1)	21.2 (17.0–26.2)	364 (92.4)	20.5 (16.6–25.0)	252 (92.3)	13.9 (10.1–18.8)
No information	208 (–)	–	69 (–)	–	72 (–)	–	67 (–)	–
Diarrhea								
Yes	88 (8.6)	13.8 (7.8–23.1)	28 (8.2)	7.1 (1.8–24.6)	34 (8.6)	21.9 (10.8–39.4)	26 (9.5)	11.5 (3.7–30.4)
No	922 (91.4)	20.3 (17.7–23.2)	312 (91.8)	22.3 (18.0–27.3)	361 (91.4)	20.1 (16.3–24.6)	249 (90.5)	14.9 (10.9–20.0)
No information	204 (–)	–	68 (–)	–	71 (–)	–	65 (–)	–
Dyspnea								
Yes	89 (8.8)	15.6 (9.1–25.5)	26 (7.7)	20.0 (8.5–40.1)	38 (9.7)	13.2 (5.6–28.0)	25 (9.2)	12.5 (4.1–32.5)
No	910 (91.2)	20.2 (17.5–23.1)	311 (92.3)	21.4 (17.1–26.3)	353 (90.3)	21.3 (17.3–25.9)	246 (90.8)	13.6 (9.8–18.6)
No information	215 (–)	–	71 (–)	–	75 (–)	–	69 (–)	–
Sore throat								
Yes	404 (37.6)	17.4 (13.8–21.6)	133 (37.1)	18.2 (12.5–25.7)	153 (37.1)	16.6 (11.4–23.4)	118 (40.5)	17.4 (11.5–25.5)
No	659 (62.4)	20.9 (17.8–24.4)	226 (62.9)	23.2 (18.1–29.2)	260 (62.9)	21.8 (17.1–27.3)	173 (59.5)	11.3 (7.3–17.1)
No information	151 (–)	–	49 (–)	–	53 (–)	–	49 (–)	–
Shortness of breath								
Yes	126 (12.6)	14.2 (8.8–21.9)	46 (13.4)	19.6 (10.5–33.6)	49 (12.2)	8.2 (3.1–19.9)^a^	31 (11.2)	13.3 (5.1–30.7)
No	894 (87.4)	20.3 (17.7–23.3)	296 (86.6)	21.2 (16.8–26.2)	353 (87.8)	21.6 (17.5–26.2)	245 (88.8)	14.6 (10.7–19.7)
No information	194 (–)	–	66 (–)	–	64 (–)	–	64 (–)	–

^a^ p-value < 0.05.

About 30% analyzed students had contact with suspected or confirmed covid-19 cases. Seroprevalence of SARS-CoV-2 antibodies was higher among those who reported contact with covid-19 cases, in the municipal public school system (29.7%; 95%CI 24.2–35.8; p<0.001), and private school system (20.3%, 95%CI15.4–26.2; p = 0.027) (Table 3).

**Table 3:** Distribution and seroprevalence of SARS-CoV-2 antibodies in schoolchildren according to risk factors, recommended measures, and school system. City of São Paulo, 2020.

Variables	TOTAL		Municipal public school system		State public school system		Private school system	
			
	n (%)	Seroprevalence (95%CI)	n (%)	Seroprevalence (95%CI)	n (%)	Seroprevalence (95%CI)	n (%)	Seroprevalence (95%CI)
Contact with suspected or confirmed case								
Yes	730 (28.6)	24.3 (21.2–27.8)^a^	239 (27.9)	29.7 (24.2–35.8)^a^	274 (28.6)	20.3 (15.9–25.5)	217 (30.5)	20.3 (15.4–26.2)^a^
No	1,717 (68.1)	15.3 (13.6–17.2)	589 (68.6)	15.0 (12.3–18.2)	649 (67.7)	17.9 (15.1–21.1)	479 (67.3)	9.6 (7.2–12.6)
Uninformed	82 (3.3)	21.7 (13.6–32.7)	30 (3.5)	31.0 (17.0–49.7)	35 (3.7)	14.3 (6.1–30.1)	16 (2.2)	12.5 (3.1–38.6)
No information	1,669 (–)	–	610 (–)	–	563 (–)	–	496 (–)	–
Social distancing								
Total	2,855 (76.6)	15.3 (13.9–16.8)^a^	955 (74.7)	17.2 (14.9–19.8)^a^	1,038 (76.7)	14.7 (12.7–17.1)^a^	862 (81.5)	11.5 (9.5–13.9)
Partial	795 (22.2)	21.1 (18.3–24.3)	304 (23.8)	22.9 (18.5–28.0)	301 (22.3)	21.2 (16.9–26.2)	189 (17.8)	14.5 (10.1–20.3)
No social distancing	40 (1.2)	29.6 (17.1–46.0)	19 (1.5)	36.8 (18.7–59.7)	14 (1.0)	21.4 (7.1–49.4)	7 (0.7)	14.3 (2.0–58.0)
No information	508 (–)	–	190 (–)	–	168 (–)	–	150 (–)	–
Use of a face mask								
Always	3,447 (81.7)	16.1 (14.8–17.4)	1,183 (80.7)	17.7 (15.6–20.0)	1,226 (80.9)	16.3 (14.3–18.5)	1,037 (86.4)	11.5 (9.7–13.7)
Most of times	454 (11.2)	17.9 (14.5–22.0)	176 (12.0)	22.3 (16.7–29.0)	167 (11.0)	13.4 (9.0–19.5)	111 (9.2)	14.5 (9.1–22.4)
Sometimes	185 (4.8)	22.3 (16.8–29.1)	75 (5.1)	24.0 (15.7–34.9)	82 (5.4)	21.9 (14.3–32.2)	28 (2.3)	14.3 (5.5–32.4)
Never	98 (2.3)	23.8 (10.1–46.5)	32 (2.2)	40.0 (15.8–70.3)	41 (2.7)	12.5 (4.8–28.9)	25 (2.1)	20.0 (2.7–69.1)
No information	14 (–)	–	2 (–)	–	5 (–)	–	7 (–)	–
Use of public transportation								
No	2,845 (66.5)	15.2 (13.8–16.6)^a^	937 (64.5)	17.1 (14.9–19.7)	956 (63.4)	15.2 (13.0–17.6)	952 (79.7)	10.5 (8.7–12.7)^a^
Sometimes	1,252 (32.0)	19.0 (15.9–21.4)	492 (33.9)	21.1 (17.7–25.0)	527 (35.0)	17.1 (14.1–20.6)	232 (19.4)	17.2 (12.8–22.6)
Frequently	58 (1.5)	22.9 (13.6–36.0)	24 (1.6)	21.7 (9.4–42.8)	24 (1.6)	25.0 (11.7–45.6)	10 (0.9)	20.0 (5.0–54.0)
No information	43 (–)	–	15 (–)	–	14 (–)	–	14 (–)	–
Coexistence with person over 60 years of age								
Yes	1,192 (28.2)	14.6 (12.6–19.9)	398 (27.3)	18.4 (14.9–22.6)	426 (28.3)	13.3 (10.4–16.9)	368 (30.7)	8.0 (5.6–11.3)^a^
No	2,970 (71.8)	17.1 (15.7–18.6)	1,058 (72.7)	18.4 (16.2–20.9)	1,081 (71.7)	17.1 (14.9–19.5)	830 (69.3)	13.3 (11.2–15.9)
No information	36 (–)	–	12 (–)	–	14 (–)	–	10 (–)	–
Existence of a resident who works outside the home								
Yes	3,376 (81.2)	16.7 (15.4–18.1)	1,199 (82.1)	18.1 (16.0–20.4)	1,219 (80.7)	16.7 (14.7–18.9)	957 (79.8)	12.9 (10.9–15.2)^a^
No	794 (18.8)	15.5 (12.9–18.4)	261 (17.9)	20.5 (16.0–25.8)	291 (19.3)	13.3 (9.9–17.8)	242 (20.2)	7.7 (4.9–11.8)
No information	28 (–)	–	8 (–)	–	11 (–)	–	9 (–)	–
Place where the person stayed most of the time								
In their own home	3,783 (90.1)	16.1 (14.9–17.4)^a^	1,317 (89.9)	17.8 (15.8–20.0)^a^	1,352 (89.2)	15.9 (14.1–18.0)	1,113 (92.7)	11.6 (9.9–13.7)
Family/neighbor’s home	399 (9.9)	20.7 (16.8–25.2)	148 (10.1)	25.2 (18.8–32.8)	163 (10.8)	17.4 (12.3–24.0)	88 (7.3)	14.1 (8.2–23.2)
No information	16 (–)	–	3 (–)	–	6 (–)	–	7 (–)	–

^a^ p-value < 0.05.

Regarding the practice of social distancing by the students, more than 75% reported practicing it completely and more than 80% declared wearing a face mask regularly. In municipal and state public school systems, seroprevalence of SARS-CoV-2 antibodies was higher among those who did not adopt social distancing (36.8%; 95%CI 18.7–59.7; p = 0.012 and 21.4 %; 95%CI 7.1–49.4; p = 0.026, respectively). In the private school system, seroprevalence was higher among those who used public transportation (20.0%; 95%CI 5.0–54.0; p = 0.015) and lower among those who did not live with someone who worked outside the home (7.7%; 95%CI 4.9–11.8; p = 0.027) (Table 3).

During the study period, about 30% students interviewed coexisted with someone over 60 years of age in the same housing unit; in the private system, seroprevalence of SARS-CoV-2 antibodies was lower among students who coexisted with older people (8.0%; 95%CI 5.6–11.3, p = 0.008) (Table 3).

Most students, around 90%, stayed at their own home during school closure. However, in the municipal public school system, seroprevalence was higher among those who stayed with family members or neighbors (25.2%; 95%CI 18.8–32.8; p = 0.030) (Table 3).

## DISCUSSION

This study estimated seroprevalence of total SARS-CoV-2 antibodies in the city of São Paulo and its association with clinical, socioeconomic, and demographic characteristics, as well as measures against covid-19 in students from private and municipal and state public school systems during school closure. The results obtained showed higher seroprevalence among black and brown students, in the most vulnerable social class (lower class), in the most peripheral city regions, and among those who reported not practicing preventive measures against covid-19.

Seroprevalence found in this study was of 16.6% (95%CI 15.4–17.8), while in the study by Pinto Junior et al.^16^ seroprevalence in schoolchildren was of 28.0%, with sample collection carried out in November and early December 2020 using the Leccurate SARS-CoV-2 Antibody Rapid Test Kit (Colloidal Gold Immunochromatography) in a distal tip sample in the city of Fortaleza. In the household survey carried out in England and Wales, in which a cohort of children was observed weekly through online interviews, seroprevalence ranged from 9.8 % to 13.0% according to age group^17^. In the systematic review and meta-analysis carried out by Badal et al.^3^, prevalence of 21% (95%CI 16–25) was found in children aged 2 to 6 years; 25% (95%CI 18–32) from 6 to 10 years; 23% (95%CI 17–25) in children aged 11 to 14 years; and 15% (CI95 8–24) in those aged 8 years and over. In the health survey carried out in 2020 in the United States, seroprevalence in children and adolescents under 19 years of age was of 8.5% (95%CI 6.9–10.3), with the highest prevalence in white people (8.2%; 95%CI 6.4–10.5) and in children younger than 5 years (13.7%; 95%CI 9.5–19.5), using the Ortho Clinical test, which detects total antibodies^18^. A seroprevalence study carried out in Zurich, Switzerland, using the SenASTrIS test, with participation of 2,484 students from 55 educational institutions, found seroprevalence of 2.8% (95%CI 1.5–4.1)19. In China, one estimated seroprevalence of 6.2% in children under 14 years of age and 8.6% in people aged between 15 and 64 years. The increase in seroprevalence according to age group was similar to that found in surveys carried out in England, Italy, Japan, Singapore, Canada, the United States, and South Korea^2^.

Nonetheless, the comparison between the results of different studies should be done with caution, since seroprevalence depends both on the moment of the pandemic evolution in which the research was developed and on the laboratory test used.

The highest seroprevalence in the black or brown people and in the most vulnerable social class was similar to that found in several studies^20^. In Brazil, the serological survey in adults carried out by the same research team of this study in the city of São Paulo^12^ and the study by Barros et al.^21^ showed this disparity as well.

Supporting the studies by Rocha et al.^22^and Martins-Filho et al.^23^, covid-19 was associated with issues of socioeconomic vulnerability rather than with population demographic characteristics. Disparities in health determinants, in the access to and quality of health services, and in sanitary and housing conditions increase the risk of diseases that can affect the population health.

Among those who tested positive for total SARS-CoV-2 antibodies and who had symptoms, the most reported clinical signs were runny nose, cough, sore throat, headache, and fever, which is in line with reports from other studies^3,5, 16^.

Seroprevalence of SARS-CoV-2 antibodies was also associated with contact with a suspected or confirmed covid-19 case, data consistent with studies carried out in Mexico^24^ and China^8^. Contact tracing using serological tests to detect individuals infected by the virus and assessing the disease prevalence in the community are essential for understanding the disease epidemiological situation in all age groups^25^.

Studies show that social distancing and adoption of protective measures against covid-19 reduce contact between people and the possibility of contracting the disease by shortening the virus transmission chain^26^.

With school closure, it was necessary for children to stay at home, often under the care of an adult, causing an increase in parental absenteeism at work, or stay with grandparents, which can increase the risk in this group^27^. In this study, the proportion of students who coexisted with older people in the same housing unit represented almost 30% cases.

The interpretation of study findings should consider some limitations. Difficulties in sample collection and interviews with students were expected and then tried to be compensated by a 50% increase in the sample size in order to obtain the fixed precision. The main reasons for nonresponse were: impossibility of identifying the address or household selected on the database; those responsible for the student refused to meet the research team or the student refused to participate in the study; difficulty for the research team to obtain permission to enter condominiums, and absence of the selected student or person responsible for the child in the household. Other studies of the same nature have pointed out that failures in the databases and in the use of location instruments, fear of meeting the team, and difficulty in collecting samples from children can be impediments to carrying out field research^28^. This study maintained good accuracy in estimating seroprevalence of SARS-CoV-2 antibodies in schoolchildren, with a sample error of around 3%. The sample size actually collected was greater than the minimum sample size calculated. Another limitation is that the Wondfo SARS-CoV-2 Antibody Test^®^ (lateral flow method) which detects total SARS-CoV-2 antibodies (IgG/IgM) used for venous blood sample collection does not allow determining whether the infection is current. In addition, serological tests can generate false negative results in recent infections^29^.

The study on seroprevalence of SARS-CoV-2 antibodies in children should be monitored even in places of low transmission in order to recommend protective measures against the virus. The disease transmission depends on the moment in which the studies are carried out, and, therefore, festive dates, school holidays, and the reduction of restriction measures should be considered^30^.

Considering the high virus transmission capacity and the importance of the school for the children’s educational, social, mental, and physical development and the reduction of social, economic, and cultural inequalities in the population, non-pharmacological prevention measures and monitoring of the serological students’ status are essential for a proper return to face-to-face classes, since there is a greater difficulty in following rules and hygiene in these age groups, especially regarding the population in greater social vulnerability and that have not yet been included in the vaccination schedule.
