# Supplementary material for: Seroprevalence of SARS-CoV-2 antibodies in schoolchildren in the city of São Paulo, 2020
Source: Rev Saude Publica. 2023 May 11;57(Suppl 1):6s. doi: 10.11606/s1518-8787.2023057004782 (PMC10185315; doi:10.11606/s1518-8787.2023057004782)
Supplement: Translation

# Translation: Soroprevalência de anticorpos do vírus SARS-CoV-2 em escolares no município de São Paulo, 2020

**Authors:** Gabriela Akemi Kamioka, Geraldine Madalosso, José Olimpio Moura de Albuquerque, Selma Anequini Costa, Paula Bisordi Ferreira, Ana Paula Sayuri Sato, Paula Regina Glasser, Francisco Alberto Pino, Patrícia Carla Piragibe Ramos Burihan, Ana Carolina Aguiar de Carvalho, Ana Beatriz Pagliaro Amorim, Cinthya Luzia Cavazzana, Caroline Cotrim Aires, Ana Paula Arruda Geraldes Kataoka, Elisa San Martin Mouriz Savani, Thirsa Alvares Franco Bessa, Breno Souza de Aguiar, Marcelo Antunes Failla, Edson Aparecido dos Santos, Edjane Maria Torreão Brito, Maria Cristina Honório dos Santos, Luiz Artur Vieira Caldeira, Solange Maria Saboia e Silva, Luiz Carlos Zamarco, Sandra Maria Sabino Fonseca, Marcia Maria de Cerqueira Lima, Ivanilda Argenau Marques, Athenê Maria de Marco França Mauro, Eduardo de Masi

**Affiliations:** I Brasil Secretaria Municipal de Saúde. Coordenadoria de Vigilância em Saúde. São Paulo, SP, Brasil; II Brasil Universidade de São Paulo. Faculdade de Saúde Pública. São Paulo, SP, Brasil; III Brasil Secretaria de Agricultura e Abastecimento. Instituto de Economia Agrícola. São Paulo, SP, Brasil; IV Brasil Secretaria Municipal de Saúde. São Paulo, SP, Brasil

**Keywords:** Criança, Adolescente, COVID-19, epidemiologia, SARS-CoV-2, Estudos Soroepidemiológicos

## Abstract

**OBJETIVO:**

Estimar a soroprevalência de anticorpos do vírus SARS-CoV-2 em escolares de quatro a 14 anos de idade residentes no município de São Paulo, segundo variáveis clínicas, demográficas, epidemiológicas e sociais, durante o período de fechamento das escolas como medida de controle da covid-19.

**MÉTODOS:**

Realizou-se um inquérito sorológico em setembro de 2020 com amostra aleatória estratificada por tipo de rede de ensino (pública municipal, pública estadual e privada). Foi coletada amostra de sangue venoso utilizando-se o teste de imunoensaio de fluxo lateral da fabricante *Wondfo* para detecção de anticorpos totais contra o vírus SARS-CoV-2. Aplicaram-se questionários semiestruturados para o levantamento de dados clínicos, demográficos, sociais e epidemiológicos.

**RESULTADOS:**

A soroprevalência de anticorpos do vírus SARS-CoV-2 em escolares foi de 16,6% (IC95% 15,4–17,8). O estudo encontrou soroprevalências mais elevadas na rede pública municipal (18,5%; IC95% 16,6–20,6) e estadual (16,2%; IC95% 14,4–18,2) em relação à rede privada (11,7; IC95% 10,0–13,7) e entre escolares da raça/cor preta e parda (18,4%; IC95% 16,8–20,2) e no estrato social mais vulnerável (18,5%; IC95% 16,9–20,2). A pesquisa identificou menores soroprevalências nos escolares que relataram seguir as medidas recomendadas de proteção contra a covid-19.

**CONCLUSÃO:**

A soroprevalência de anticorpos contra o vírus SARS-CoV-2 atinge principalmente os escolares socialmente mais vulneráveis. Este estudo pode contribuir para embasar políticas públicas que reforcem a importância da suspensão das aulas presenciais e da necessidade de estratégias de medidas de proteção e acompanhamento do status sorológico daqueles que ainda não foram contemplados no calendário vacinal.

## INTRODUÇÃO

A Organização Mundial da Saúde (OMS) declarou que a covid-19, doença causada pelo vírus SARS-CoV-2, assumiu caráter pandêmico em março de 2020^1^. Considerados desafios para a saúde pública no mundo, a epidemiologia, a caracterização clínica da doença, o cuidado e a evolução dos casos de covid-19 têm sido áreas de estudo bastante demandadas. A doença afetou todas as faixas etárias em diferentes proporções e graus de manifestações clínicas^2^. Estudos mostram que as crianças apresentam sintomas mais leves e prognóstico mais favorável quando comparadas às demais faixas etárias^3^.

A idade tem sido um importante fator associado à gravidade e à mortalidade da doença^4^. Nos primeiros meses da pandemia apenas 2,1% dos casos de covid-19 foram notificados em menores de 18 anos e não foram reportados óbitos em menores de 9 anos. Dentre os que foram notificados, cerca de 4,4% apresentaram a forma grave da doença^5^ e aproximadamente 1,0% foi hospitalizado ou a óbito. Estudos mostraram soroprevalência de anticorpos do SARS-CoV-2 em crianças de até 14,6% em países da Europa e 75,2% nos Estados Unidos^6,7^. Diferenças na distribuição, maturação e funcionalidade dos receptores da enzima conversora de angiotensina 2 (ECA2) e nível elevado de linfócitos no sangue das crianças, principalmente das células *natural killer*, podem estar presentes em nível superior quando comparados aos adultos^8^.

A suspensão das aulas presenciais, com o consequente fechamento das escolas entre os anos de 2020 e 2021, foi uma medida adotada em quase todas as partes do mundo para prevenir e conter a disseminação da covid-19^9^. Ela causou significativo impacto social e econômico na sociedade em todos os seus níveis: regional, nacional e global^10^. No Brasil, em março de 2020, foram implementadas ações para o distanciamento social, com substituição de aulas presenciais por remotas, quarentena voluntária e fechamento de serviços não essenciais.

Em maio de 2020, quando houve a reabertura gradativa das escolas e unidades de ensino no município de São Paulo, o conhecimento da soroprevalência de anticorpos do vírus SARS-CoV-2 em crianças e adolescentes foi de extrema importância para a tomada de decisões seguras sobre a reabertura de serviços e unidades de ensino e mitigação da transmissão dessa doença^11^.

Em junho de 2020, a Secretaria Municipal da Saúde de São Paulo iniciou a realização do inquérito sorológico para SARS-CoV-2 em adultos^12^ e em continuidade às ações de vigilância epidemiológica, em setembro, foi realizado o inquérito sorológico em escolares. Este estudo utilizou dados do inquérito em escolares e teve como objetivo estimar a soroprevalência de anticorpos totais do vírus SARS-CoV-2 em escolares de quatro a 14 anos de idade, residentes no município de São Paulo, em 2020, segundo variáveis clínicas, demográficas, epidemiológicas e sociais.

## MÉTODOS

Trata-se de um inquérito sorológico em escolares de quatro a 14 anos de idade, residentes no município de São Paulo.

### Área do Estudo

São Paulo é a cidade mais populosa do Brasil e tem uma população estimada, em 2019, de 11,8 milhões de habitantes, sendo 1,6 milhão com idade entre quatro e 14 anos. O município apresenta grande disparidade econômica e social, refletindo heterogeneidade nas condições de educação, renda e habitação. O índice de desenvolvimento humano (IDH) das áreas de abrangência das 472 unidades básicas de saúde varia de 0,62 (com 147 áreas de unidades no intervalo de IDH de 0,62 a 0,73) a 0,95 (com 83 áreas de unidades no intervalo de IDH de 0,83 a 0,95). O município, capital do estado de São Paulo, está dividido em seis regiões de saúde, as quais também têm grande variação do IDH. As áreas de unidades de saúde com os menores valores de IDH se concentram nas regiões Sul, Leste e Norte, localizadas nos extremos do município, enquanto os maiores valores estão centralizados nas regiões Sudeste, Centro e Oeste, áreas mais próximas ao centro do município. Portanto, a desigualdade social na cidade segue um padrão concêntrico, com a população mais vulnerável tendendo às periferias e a população mais abastada ao centro, em direção à região Oeste^13^.

### População do Estudo

Os participantes que compuseram a amostra foram escolares cadastrados no banco de dados das redes públicas (municipal e estadual) e privada das Secretarias Municipal e Estadual de Educação de São Paulo, considerando os seguintes critérios de inclusão: crianças com quatro a 14 anos de idade residentes no município de São Paulo.

### Desenho Amostral

Foi realizada uma amostragem aleatória^14^, estratificada segundo rede de ensino, sendo cada rede escolar um domínio amostral (rede pública municipal, rede pública estadual e rede privada).

Para as redes municipal e estadual, o tamanho mínimo da amostra foi planejado para se obter estimativas de prevalência de 16%, com coeficiente de variação inferior a 15%, ao nível de confiança de 95%. Para a rede privada, a amostra foi calculada para se obter estimativa de prevalência de 9%, também com coeficiente de variação inferior a 15% e ao nível de confiança de 95%. Dessa forma, chegou-se ao tamanho amostral mínimo de 900 escolares para cada uma das redes públicas e de 1.200 escolares para a rede privada. Para o sorteio dos escolares, houve uma compensação para a taxa de não resposta esperada de 50%.

### Testagem e Entrevista

Realizou-se a coleta de dados entre os dias 1 e 16 de setembro de 2020 no domicílio da criança sorteada, uma vez que as aulas presenciais estavam suspensas no período de realização do estudo. A entrevista foi feita com a criança e um responsável presente no momento da visita, por meio de um formulário de coleta de dados semiestruturado, que compreendia questões sobre informações socioeconômicas, demográficas, epidemiológicas e de medidas de prevenção da doença. Os dados coletados foram incluídos em formulário eletrônico padronizado na plataforma FormSUS/DATASUS, versão 3.0, pela unidade de saúde responsável.

No estudo, para coleta de amostra de sangue venoso (soro), foi utilizado o teste de imunoensaio de fluxo lateral que detecta anticorpos totais (IgG/IgM) contra o vírus SARS-CoV-2 no sangue total, soro e plasma humanos, SARS-CoV-2 Antibody test^®^, da fabricante Guangzhou Wondfo Biotech Co., Ltd., com sensibilidade de 86,4% e especificidade de 99,6%. No Brasil, o Ministério da Saúde disponibilizou o mesmo teste com o nome One Step Covid-2019 Test^®^, por meio da empresa representante legal da fabricante Celer Biotecnologia S/A^15^.

### Análise de Dados

As soroprevalências de anticorpos do vírus SARS-CoV-2 ponderadas pelo total de coletas realizadas por estrato e os respectivos intervalos de 95% de confiança (IC95%) foram estimados de acordo com o desenho amostral usando os pacotes estatísticos R e Stata versão 13.

As estimativas de soroprevalência e respectivos IC95% foram obtidos para cada estrato amostral e segundo região de saúde, sexo, raça/cor, escolaridade dos responsáveis, estrato social, número de moradores no domicílio, faixa etária dos escolares, presença ou ausência dos seguintes sintomas no momento da entrevista: perda de olfato, perda de paladar, febre, fraqueza, tosse, cefaleia, coriza, náusea, diarreia, dispneia, dor de garganta, falta de ar; medidas tomadas durante a pandemia, local onde permaneceu a maior do tempo, se em casa ou na casa de familiar e/ou vizinho, se permaneceu na mesma residência com pessoas acima de 60 anos, se praticou distanciamento social, se na residência havia morador que trabalhava fora de casa, se teve contato com caso suspeito ou confirmado de covid-19, se usou transporte coletivo e máscara.

O estrato social foi classificado de acordo com a renda familiar informada pelo entrevistado. Foi considerado estrato alto aquele em que a renda era maior que R$ 8.640,00; estrato médio aquele em que a renda era entre R$ 2.005,00 e R$ 8.640,00; e estrato baixo aquele em que a renda era menor que R$ 2.005,00.

Para a comparação das frequências entre as categorias de cada variável, utilizou-se o teste qui-quadrado com correção de Rao Scott, considerando um nível de significância de 5%.

Para a análise da distribuição espacial da soroprevalência de anticorpos do vírus SARS-CoV-2 em escolares no município de São Paulo, foi utilizado o software *Quantum Geographic Information System* (QGIS) versão 3.22.

### Aspectos Éticos

O estudo foi aprovado pela Comissão Nacional de Ética em Pesquisa (CAAE 36032820.3.0000.0008). Os responsáveis pelos escolares selecionados para pesquisa foram devidamente informados sobre os objetivos do estudo, os riscos, os benefícios e o direito à recusa. A coleta de amostra de sangue e a entrevista somente foram realizadas após assinatura do termo de consentimento livre e esclarecido pelo responsável e do termo de assentimento pelo escolar menor de 18 anos de idade.

## RESULTADOS

Participaram do estudo 4.198 escolares, com idade entre quatro e 14 anos, residentes no município de São Paulo, sendo 1.469 da rede municipal, 1.521 da rede estadual e 1.208 da rede privada.

A soroprevalência de anticorpos totais contra o vírus SARS-CoV-2 foi de 16,6% (IC95% 15,4–17,8), sendo de 18,5% (IC95% 16,6–20,6) na rede municipal, 16,2% (IC95% 14,4–18,2) na rede estadual e 11,7% (IC95% 10,0–13,7) na rede privada. Houve diferença estatística entre as soroprevalências nos estratos educacionais (p < 0,001).

Segundo a região do município, as estimativas de soroprevalências foram diferentes apenas para a rede privada (p = 0,003), com menor soroprevalência na região Sudeste (5,8%; IC95% 3,4–9,5) e maior na região Sul (16,9%; IC95% 13,1–21,4) (Tabela 1).

**Tabela 1:** Distribuição e soroprevalência de anticorpos contra o vírus SARS-CoV-2 de escolares segundo fatores demográficos e estrato escolar. Município de São Paulo, 2020.

Variáveis demográficas	TOTAL		Rede pública municipal		Rede pública estadual		Rede privada	
			
	n (%)	Soroprevalência (IC95%)	n (%)	Soroprevalência (IC95%)	n (%)	Soroprevalência (IC95%)	n (%)	Soroprevalência (IC95%)
Total	4.198 (100,0)	16,6 (15,4–17,8)^a^	1.469 (45,7)	18,5 (16,6–20,6)	1.521 (38,2)	16,2 (14,4–18,2)	1.208 (16,1)	11,7 (10,0–13,7)
Região								
Centro-oeste	294 (6,3)	18,1 (13,7–23,5)^a^	92 (6,3)	22,2 (14,8–32,0)	67 (4,4)	18,2 (10,6–29,3)	135 (11,2)	11,5 (7,0–18,1)^a^
Norte	848 (20,1)	18,2 (15,6–21,1)	307 (20,9)	18,9 (14,8–23,7)	281 (18,5)	20,6 (16,2–25,7)	259 (21,4)	11,4 (8,1–15,9)
Sudeste	727 (16,6)	12,4 (10,0–15,2)	213 (14,5)	13,8 (9,8–19,2)	264 (17,4)	14,2 (10,4–19,1)	250 (20,7)	5,8 (3,4–9,5)
Leste	1.094 (27,3)	16,6 (14,4–19,1)	446 (30,4)	19,6 (16,2–23,6)	405 (26,6)	14,1 (11,1–17,9)	243 (20,1)	11,6 (8,1–16,3)
Sul	1.235 (29,7)	17,4 (15,3–19,7)	410 (27,9)	18,7 (15,1–22,8)	504 (33,1)	16,3 (13,3–19,8)	321 (26,6)	16,9 (13,1–21,4)
Sexo								
Masculino	2.071 (49,7)	17,9 (16,2–19,7)	743 (50,6)	19,9 (17,2–23,0)	739 (48,8)	17,3 (14,7–20,2)	589 (49,1)	13,3 (10,8–16,3)
Feminino	2.113 (50,3)	15,3 (13,7–17,0)	724 (49,4)	17,1 (14,5–20,0)	777 (51,2)	15,2 (12,8–17,9)	611 (50,9)	10,4 (8,2–13,1)
Sem informação	14 (–)	–	1 (–)	–	5 (–)	–	8 (–)	–
Faixa etária								
4 e 5 anos	954 (21,8)	16,8 (14,4–19,5)	393 (27,4)	18,0 (14,5–22,1)	139 (9,3)	16,7 (11,3–23,8)	421 (35,5)	14,3 (11,3–18,0)
6 a 10 anos	1.875 (45,6)	16,5 (14,8–18,4)	561 (39,2)	20,3 (17,2–23,9)	817 (54,7)	15,2 (12,9–17,9)	497 (41,9)	10,3 (7,9–13,3)
11 a 14 anos	1.284 (32,6)	15,9 (13,9–18,1)	478 (33,4)	16,3 (13,2–19,9)	538 (36,0)	17,0 (14,0–20,4)	268 (22,6)	10,2 (7,1–14,4)
Sem informação	85 (–)	–	36 (–)	–	27 (–)	–	22 (–)	–
Raça/cor								
Branca	2.046 (46,9)	14,5 (12,9–16,3)^a^	654 (44,8)	16,9 (14,2–20,0)	648 (42,9)	15,2 (12,6–18,2)	431 (36,2)	8,5 (6,7–10,8)^a^
Preta/Parda	2.082 (52,4)	18,4 (16,8–20,2)	796 (54,5)	19,7 (17,1–22,7)	854 (56,6)	17,1 (14,7–19,8)	744 (62,4)	17,6 (14,3–21,6)
Outras	35 (0,7)	18,2 (7,6–37,3)	10 (0,7)	30,0 (10,0–62,4)	8 (0,5)	12,5 (1,7–53,7)	17 (1,4)	6,3 (0,9–33,5)
Sem informação	35 (–)	–	8 (–)	–	11 (–)	–	16 (–)	–
Nº moradores no domicílio								
1 a 2	175 (4,5)	16,8 (14,4–19,5)	58 (4,3)	15,8 (8,4–27,7)	60 (4,3)	10,2 (4,6–20,8)	57 (5,3)	12,7 (6,2–24,4)
3 a 4	2.333 (59,3)	16,5 (14,8–18,4)	745 (56,0)	17,7 (15,1–20,6)	813 (58,3)	17,0 (14,5–19,7)	774 (71,3)	12,0 (9,9–14,5)
5 ou mais	1.303 (36,2)	15,9 (13,9–18,1)	528 (39,7)	21,1 (17,8–24,8)	521 (37,4)	15,4 (12,5–18,8)	254 (23,4)	11,3 (7,9–15,9)
Sem informação	387 (–)	–	137 (–)	–	127 (–)	–	123 (–)	–
Estrato social								
Alto	156 (2,4)	5,2 (2,4–11,0)^a^	12 (0,8)	8,3 (1,2–41,3)	6 (0,4)	16,7 (2,3–63,1)^a^	138 (11,6)	3,6 (1,5–8,4)^a^
Médio	1.379 (30,9)	12,6 (10,8–14,6)	403 (27,7)	15,4 (12,1–19,3)	429 (28,4)	11,1 (8,4–14,4)	388 (32,5)	10,1 (7,8–12,9)
Baixo	2.275 (58,6)	18,5 (16,9–20,2)	924 (63,4)	19,6 (17,2–22,3)	963 (63,9)	17,3 (15,0–19,9)	546 (45,8)	17,7 (14,2–21,9)
Não soube informar	348 (8,1)	19,2 (15,1–24,1)	118 (8,1)	20,2 (13,8–28,5)	110 (7,3)	22,4 (15,5–31,3)	120 (10,1)	11,3 (6,7–18,5)
Sem informação	40 (–)	–	11 (–)	–	13 (–)	–	16 (–)	–
Escolaridade do morador mais instruído								
Não estudou	15 (0,4)	34,5 (14,8–61,4)^a^	3 (0,2)	33,3 (4,3–84,7)	8 (0,6)	37,5 (12,5–71,5)^a^	4 (0,3)	25,0 (3,4–76,2)^a^
Fundamental	345 (9,3)	23,1 (18,9–28,1)	138 (9,9)	21,7 (15,6–29,4)	156 (10,9)	24,5 (18,3–32,0)	51 (4,4)	24,5 (14,5–38,3)
Médio	2.266 (60,0)	18,1 (16,5–19,8)	904 (64,7)	19,7 (17,2–22,4)	916 (63,7)	16,7 (14,4–19,3)	665 (57,0)	15,8 (12,7–19,6)
Superior	1,375 (30,3)	11,3 (9,6–13,3)	352 (25,2)	14,8 (11,4–18,9)	357 (24,8)	10,3 (7,5–13,9)	446 (38,3)	8,1 (6,3–10,5)
Sem informação	197 (–)	–	71 (–)	–	84 (–)	–	42 (–)	–

^a^ Valor de p < 0,05.

A Figura 1 mostra maiores soroprevalências de anticorpos do vírus SARS-CoV-2 em escolares nas regiões periféricas do município.

**Figura 1:** Distribuição espacial da soroprevalência de anticorpos contra o vírus SARS-CoV-2 em escolares, segundo Unidade de Vigilância em Saúde. Município de São Paulo, 2020.

Não houve diferença entre as soroprevalências em relação a sexo, faixa etária e total de moradores no domicílio. No entanto, na rede privada, as estimativas de soroprevalências na raça/cor referida preta ou parda foram significativamente maiores (17,6%; IC95% 14,3–21,6; p < 0,001). Nas redes pública estadual e privada, a soroprevalência foi maior entre os escolares que conviviam com moradores menos instruídos (24,5%; IC95% 18,3–32,0; p < 0,001 e 24,5%; IC95% 14,5–38,3; p < 0,001, respectivamente) e do estrato social mais vulnerável (17,3%; IC95% 15,0–19,9 e 17,7%; IC95% 14,2–21,9, respectivamente) (Tabela 1).

A Tabela 2 apresenta as estimativas de soroprevalência de anticorpos totais contra o vírus SARS-CoV-2 segundo variáveis clínicas. A proporção de crianças com sintomas no momento da entrevista foi de 29,1%. Os principais sintomas apresentados foram: coriza (64,1%), tosse (59,3%), dor de garganta (37,6%), cefaleia (33,2%) e febre (29,8%). Os sintomas de perda de olfato (55,0%; IC95% 38,2–70,7; p < 0,001), perda de paladar (47,3%; IC95% 33,2–61,8; p < 0,001) e febre (26,5%; IC95% 21,7–32,0) foram associados a maiores soroprevalências de anticorpos totais contra o vírus SARS-CoV-2.

**Tabela 2:** Distribuição e soroprevalência de anticorpos contra o vírus SARS-CoV-2 de escolares segundo fatores clínicos e estrato escolar. Município de São Paulo, 2020.

Variável	TOTAL		Rede pública municipal		Rede pública estadual		Rede privada	
			
	n (%)	Soroprevalência (IC95%)	n (%)	Soroprevalência (IC95%)	n (%)	Soroprevalência (IC95%)	n (%)	Soroprevalência (IC95%)
Presença de sintomas no momento da entrevista								
Sim	1.214 (29,1)	15,4 (14,0–16,8)^a^	408 (27,9)	20,7 (17,1–25,0)	466 (30,7)	19,1 (15,8–23,0)^a^	340 (28,3)	15,1 (11,6–19,3)^a^
Não	2.968 (70,9)	19,2 (17,0–21,7)	1.054 (72,1)	17,6 (15,4–20,0)	1.050 (69,3)	14,7 (12,7–17,0)	863 (71,7)	10,5 (8,6–12,8)
Sem informação	16 (–)		6 (–)	–	5 (–)	–	5 (–)	–
Procurou atendimento médico								
Sim	302 (24,8)	21,9 (17,4–27,3)	95 (24,1)	25,3 (17,5–35,0)	107 (23,6)	20,8 (14,1–29,6)	100 (30,3)	17,0 (10,8–25,7)
Não	877 (75,2)	17,9 (15,4–20,7)	300 (75,9)	19,2 (15,1–24,1)	347 (76,4)	18,0 (14,3–22,5)	230 (69,7)	13,5 (9,6–18,7)
Sem informação	35 (–)	–	13 (–)	–	12 (–)	–	10 (–)	–
Perda de olfato								
Sim	38 (3,9)	55,0 (38,2–70,7)^a^	12 (3,6)	58,3 (30,6–81,6)^a^	17 (4,4)	50,0 (27,2–72,8)^a^	9 (3,3)	62,5 (28,3–87,5)^a^
Não	955 (96,1)	18,9 (16,4–21,7)	325 (96,4)	20,5 (16,4–25,3)	369 (95,6)	19,2 (15,4–23,6)	261 (96,7)	13,3 (9,7–18,1)
Sem informação	221 (–)	–	71 (–)	–	80 (–)	–	70 (–)	–
Perda de paladar								
Sim	49 (4,9)	47,3 (33,2–61,8)^a^	14 (4,1)	42,8 (20,6–68,5)	22 (5,7)	50,0 (30,2–69,8)^a^	13 (4,7)	50,0 (24,3–75,7)^a^
Não	953 (95,1)	18,9 (16,4–21,7)	323 (95,9)	20,9 (16,8–25,8)	367 (94,3)	19,1 (15,3–23,5)	263 (95,3)	12,8 (9,3–17,5)
Sem informação	212 (–)	–	71 (–)	–	77 (–)	–	64 (–)	–
Febre								
Sim	317 (29,8)	26,5 (21,7–32,0)	105 (29,8)	26,9 (19,3–36,3)	120 (29,2)	28,0 (20,6–36,8)^a^	92 (31,7)	22,0 (14,6–31,7)
Não	737 (70,2)	17,1 (14,4–20,2)	248 (70,2)	18,7 (14,3–24,1)	291 (70,8)	17,0 (13,1–21,8)	198 (68,3)	12,9 (8,9–18,5)
Sem informação	160 (–)	–	55 (–)	–	55 (–)	–	50 (–)	–
Fraqueza								
Sim	95 (8,9)	26,5 (18,2–37,0)	25 (7,4)	28,0 (13,9–48,3)	38 (9,6)	27,0 (15,2–43,4)	32 (11,5)	22,6 (11,1–40,5)
Não	916 (91,1)	19,6 (17,0–22,5)	312 (92,6)	21,4 (17,1–26,3)	358 (90,4)	20,1 (16,2–24,6)	246 (88,5)	13,3 (9,6–18,3)
Sem informação	203 (–)	–	71 (–)	–	70 (–)	–	62 (–)	–
Tosse								
Sim	656 (59,3)	19,0 (16,0–23,8)	220 (59,9)	22,0 (17,0–28,0)	249 (58,0)	17,7 (13,5–23,0)	187 (60,9)	13,8 (9,5–19,7)
Não	447 (40,7)	19,7 (16,1–23,8)	147 (40,1)	19,9 (14,1–27,2)	180 (42,0)	21,0 (15,6–27,7)	120 (39,1)	15,3 (9,8–23,0)
Sem informação	111 (–)	–	41 (–)	–	37 (–)	–	33 (–)	–
Cefaleia								
Sim	342 (33,2)	20,6 (16,4–25,5)	117 (33,7)	23,9 (17,0–32,5)	139 (33,7)	18,8 (13,1–26,3)	86 (30,1)	15,3 (9,1–24,6)
Não	703 (66,8)	19,1 (16,2–22,3)	230 (66,3)	19,8 (15,1–25,5)	273 (66,3)	20,4 (16,0–25,7)	200 (69,9)	13,4 (9,3–19,0)
Sem informação	169 (–)	–	61 (–)	–	54 (–)	–	54 (–)	–
Coriza								
Sim	723 (64,1)	17,5 (14,7–20,6)	249 (65,9)	18,6 (14,2–24,0)	257 (60,1)	18,8 (14,5–24,1)	217 (69,3)	11,3 (7,7–16,4)
Não	396 (35,9)	21,7 (17,7–26,3)	129 (34,1)	25,0 (18,2–33,3)	171 (39,9)	19,6 (14,3–26,4)	96 (30,7)	18,3 (11,7–27,5)
Sem informação	95 (–)	–	30 (–)	–	38 (–)	–	27 (–)	–
Náusea								
Sim	81 (8,2)	21,3 (13,3–32,4)	30 (8,9)	26,7 (13,9–45,1)	30 (7,6)	17,2 (7,3–35,4)	21 (7,7)	14,3 (4,6–36,3)
Não	925 (91,8)	19,8 (17,2–22,7)	309 (91,1)	21,2 (17,0–26,2)	364 (92,4)	20,5 (16,6–25,0)	252 (92,3)	13,9 (10,1–18,8)
Sem informação	208 (–)	–	69 (–)	–	72 (–)	–	67 (–)	–
Diarreia								
Sim	88 (8,6)	13,8 (7,8–23,1)	28 (8,2)	7,1 (1,8–24,6)	34 (8,6)	21,9 (10,8–39,4)	26 (9,5)	11,5 (3,7–30,4)
Não	922 (91,4)	20,3 (17,7–23,2)	312 (91,8)	22,3 (18,0–27,3)	361 (91,4)	20,1 (16,3–24,6)	249 (90,5)	14,9 (10,9–20,0)
Sem informação	204 (–)	–	68 (–)	–	71 (–)	–	65 (–)	–
Dispneia								
Sim	89 (8,8)	15,6 (9,1–25,5)	26 (7,7)	20,0 (8,5–40,1)	38 (9,7)	13,2 (5,6–28,0)	25 (9,2)	12,5 (4,1–32,5)
Não	910 (91,2)	20,2 (17,5–23,1)	311 (92,3)	21,4 (17,1–26,3)	353 (90,3)	21,3 (17,3–25,9)	246 (90,8)	13,6 (9,8–18,6)
Sem informação	215 (–)	–	71 (–)	–	75 (–)	–	69 (–)	–
Dor de garganta								
Sim	404 (37,6)	17,4 (13,8–21,6)	133 (37,1)	18,2 (12,5–25,7)	153 (37,1)	16,6 (11,4–23,4)	118 (40,5)	17,4 (11,5–25,5)
Não	659 (62,4)	20,9 (17,8–24,4)	226 (62,9)	23,2 (18,1–29,2)	260 (62,9)	21,8 (17,1–27,3)	173 (59,5)	11,3 (7,3–17,1)
Sem informação	151 (–)	–	49 (–)	–	53 (–)	–	49 (–)	–
Falta de ar								
Sim	126 (12,6)	14,2 (8,8–21,9)	46 (13,4)	19,6 (10,5–33,6)	49 (12,2)	8,2 (3,1–19,9)^a^	31 (11,2)	13,3 (5,1–30,7)
Não	894 (87,4)	20,3 (17,7–23,3)	296 (86,6)	21,2 (16,8–26,2)	353 (87,8)	21,6 (17,5–26,2)	245 (88,8)	14,6 (10,7–19,7)
Sem informação	194 (–)	–	66 (–)	–	64 (–)	–	64 (–)	–

^a^ Valor de p < 0,05.

Cerca de 30% dos escolares estudados tiveram contato com casos suspeitos ou confirmados de covid-19. A soroprevalência de anticorpos contra o vírus SARS-CoV-2 foi maior entre aqueles que referiram contato com casos de covid-19, na rede pública municipal (29,7%; IC95% 24,2–35,8; p < 0,001) e privada (20,3%, IC95%15,4–26,2; p = 0,027) (Tabela 3).

**Tabela 3:** Distribuição e soroprevalência de anticorpos contra o vírus SARS-CoV-2 de escolares segundo fatores de risco, medidas recomendadas e estrato escolar. Município de São Paulo, 2020.

Variáveis	TOTAL		Rede pública municipal		Rede pública estadual		Rede privada	
			
	n (%)	Soroprevalência (IC95%)	n (%)	Soroprevalência (IC95%)	n (%)	Soroprevalência (IC95%)	n (%)	Soroprevalência (IC95%)
Contato com caso suspeito ou confirmado								
Sim	730 (28,6)	24,3 (21,2–27,8)^a^	239 (27,9)	29,7 (24,2–35,8)^a^	274 (28,6)	20,3 (15,9–25,5)	217 (30,5)	20,3 (15,4–26,2)^a^
Não	1.717 (68,1)	15,3 (13,6–17,2)	589 (68,6)	15,0 (12,3–18,2)	649 (67,7)	17,9 (15,1–21,1)	479 (67,3)	9,6 (7,2–12,6)
Não soube informar	82 (3,3)	21,7 (13,6–32,7)	30 (3,5)	31,0 (17,0–49,7)	35 (3,7)	14,3 (6,1–30,1)	16 (2,2)	12,5 (3,1–38,6)
Sem informação	1.669 (–)	–	610 (–)	–	563 (–)	–	496 (–)	–
Distanciamento social								
Totalmente	2.855 (76,6)	15,3 (13,9–16,8)^a^	955 (74,7)	17,2 (14,9–19,8)^a^	1.038 (76,7)	14,7 (12,7–17,1)^a^	862 (81,5)	11,5 (9,5–13,9)
Parcialmente	795 (22,2)	21,1 (18,3–24,3)	304 (23,8)	22,9 (18,5–28,0)	301 (22,3)	21,2 (16,9–26,2)	189 (17,8)	14,5 (10,1–20,3)
Não praticou	40 (1,2)	29,6 (17,1–46,0)	19 (1,5)	36,8 (18,7–59,7)	14 (1,0)	21,4 (7,1–49,4)	7 (0,7)	14,3 (2,0–58,0)
Sem informação	508 (–)	–	190 (–)	–	168 (–)	–	150 (–)	–
Uso de máscara								
Sempre	3.447 (81,7)	16,1 (14,8–17,4)	1.183 (80,7)	17,7 (15,6–20,0)	1.226 (80,9)	16,3 (14,3–18,5)	1.037 (86,4)	11,5 (9,7–13,7)
Na maioria das vezes	454 (11,2)	17,9 (14,5–22,0)	176 (12,0)	22,3 (16,7–29,0)	167 (11,0)	13,4 (9,0–19,5)	111 (9,2)	14,5 (9,1–22,4)
De vez em quando	185 (4,8)	22,3 (16,8–29,1)	75 (5,1)	24,0 (15,7–34,9)	82 (5,4)	21,9 (14,3–32,2)	28 (2,3)	14,3 (5,5–32,4)
Nunca	98 (2,3)	23,8 (10,1–46,5)	32 (2,2)	40,0 (15,8–70,3)	41 (2,7)	12,5 (4,8–28,9)	25 (2,1)	20,0 (2,7–69,1)
Sem informação	14 (–)	–	2 (–)	–	5 (–)	–	7 (–)	–
Utilizou transporte coletivo								
Não usou	2.845 (66,5)	15,2 (13,8–16,6)^a^	937 (64,5)	17,1 (14,9–19,7)	956 (63,4)	15,2 (13,0–17,6)	952 (79,7)	10,5 (8,7–12,7)^a^
De vez em quando	1.252 (32,0)	19,0 (15,9–21,4)	492 (33,9)	21,1 (17,7–25,0)	527 (35,0)	17,1 (14,1–20,6)	232 (19,4)	17,2 (12,8–22,6)
Com frequência	58 (1,5)	22,9 (13,6–36,0)	24 (1,6)	21,7 (9,4–42,8)	24 (1,6)	25,0 (11,7–45,6)	10 (0,9)	20,0 (5,0–54,0)
Sem informação	43 (–)	–	15 (–)	–	14 (–)	–	14 (–)	–
Permaneceu com pessoa > 60 anos								
Sim	1.192 (28,2)	14,6 (12,6–19,9)	398 (27,3)	18,4 (14,9–22,6)	426 (28,3)	13,3 (10,4–16,9)	368 (30,7)	8,0 (5,6–11,3)^a^
Não	2.970 (71,8)	17,1 (15,7–18,6)	1.058 (72,7)	18,4 (16,2–20,9)	1.081 (71,7)	17,1 (14,9–19,5)	830 (69,3)	13,3 (11,2–15,9)
Sem informação	36 (–)	–	12 (–)	–	14 (–)	–	10 (–)	–
Se há morador que trabalha fora de casa								
Sim	3.376 (81,2)	16,7 (15,4–18,1)	1.199 (82,1)	18,1 (16,0–20,4)	1.219 (80,7)	16,7 (14,7–18,9)	957 (79,8)	12,9 (10,9–15,2)^a^
Não	794 (18,8)	15,5 (12,9–18,4)	261 (17,9)	20,5 (16,0–25,8)	291 (19,3)	13,3 (9,9–17,8)	242 (20,2)	7,7 (4,9–11,8)
Sem informação	28 (–)	–	8 (–)	–	11 (–)	–	9 (–)	–
Onde permaneceu na maior parte do tempo								
Na própria casa	3.783 (90,1)	16,1 (14,9–17,4)^a^	1.317 (89,9)	17,8 (15,8–20,0)^a^	1.352 (89,2)	15,9 (14,1–18,0)	1.113 (92,7)	11,6 (9,9–13,7)
Casa de familiar/vizinho	399 (9,9)	20,7 (16,8–25,2)	148 (10,1)	25,2 (18,8–32,8)	163 (10,8)	17,4 (12,3–24,0)	88 (7,3)	14,1 (8,2–23,2)
Sem informação	16 (–)	–	3 (–)	–	6 (–)	–	7 (–)	–

^a^ Valor de p < 0,05.

Sobre a prática de distanciamento social pelos escolares participantes do estudo, mais de 75% referiram praticar totalmente e mais de 80% adotaram o uso de máscara sempre. Nas redes municipal e estadual, a soroprevalência de anticorpos do vírus SARS-CoV-2 foi maior entre aqueles que não praticaram o distanciamento social (36,8%; IC95% 18,7–59,7; p = 0,012 e 21,4%; IC95% 7,1–49,4; p = 0,026, respectivamente). Na rede privada, a soroprevalência foi maior entre aqueles que utilizaram transporte público coletivo (20,0%; IC95% 5,0–54,0; p = 0,015) e menor entre aqueles que não moravam com alguém que trabalhasse fora de casa (7,7%; IC95% 4,9–11,8; p = 0,027) (Tabela 3).

Durante o período do estudo, cerca de 30% dos escolares entrevistados permaneceram com pessoa maior de 60 anos no mesmo domicílio, sendo que, na rede privada, a soroprevalência de anticorpos contra o vírus SARS-CoV-2 foi menor entre os escolares que viviam com pessoas idosas (8,0%; IC95% 5,6–11,3, p = 0,008) (Tabela 3).

A maioria dos escolares, cerca de 90%, permaneceu no próprio domicílio durante o período de fechamento das escolas. No entanto, na rede pública municipal, a soroprevalência foi mais elevada entre aqueles que ficaram na casa de familiares ou de vizinhos (25,2%; IC95% 18,8–32,8; p = 0,030) (Tabela 3).

## DISCUSSÃO

Este estudo estimou a soroprevalência de anticorpos totais contra o vírus SARS-CoV-2 no município de São Paulo e sua associação com características clínicas, socioeconômicas, demográficas e medidas adotadas para a prevenção da covid-19 em escolares da rede privada e das redes públicas, municipal e estadual, durante o período de fechamento das escolas. Os resultados obtidos mostraram maiores soroprevalências na raça/cor preta e parda, no estrato social mais vulnerável, nas regiões mais periféricas do município e entre aqueles que referiram não praticar medidas de prevenção contra a covid-19.

A soroprevalência encontrada neste estudo foi de 16,6% (IC95% 15,4–17,8), ao passo que no estudo de Pinto Junior et al.^16^ a soroprevalência em escolares foi de 28,0%, com coleta de amostras realizadas em novembro e início de dezembro de 2020 utilizando o teste de anticorpos imunocromatográfico *Leccurate* SARS-CoV-2 em amostra de ponta distal no município de Fortaleza. No inquérito domiciliar realizado na Inglaterra e no País de Gales, em que uma coorte de crianças foi observada semanalmente por meio de entrevistas on-line, a soroprevalência variou de 9,8 a 13,0% conforme a faixa etária^17^. Na revisão sistemática e metanálise realizada por Badal et al.^3^ foram encontradas prevalências de 21% (IC95% 16–25) em crianças de 2 a 6 anos; 25% (IC95% 18–32) de 6 a 10 anos; 23% (IC95% 17–25) em 11 a 14 anos; e 15% (IC95 8–24) com 8 anos ou mais. No inquérito de saúde realizado em 2020, nos Estados Unidos, a soroprevalência em crianças e adolescentes menores de 19 anos foi de 8,5% (IC95% 6,9–10,3), sendo as maiores prevalências na raça/cor branca (8,2%; IC95% 6,4–10,5) e em menores de 5 anos (13,7%; IC95% 9,5–19,5), utilizando o teste *Ortho Clinical*, que detecta anticorpos totais^18^. No estudo de soroprevalência realizado em Zurich, na Suíça, utilizando o teste *SenASTrIS*, em que participaram 2.484 alunos de 55 instituições escolares, a soroprevalência encontrada foi de 2,8% (IC95% 1,5–4,1)^19^. Na China, estimou-se soroprevalência de 6,2% em menores de 14 anos e 8,6% em pessoas com idade entre 15 e 64 anos. O aumento da soroprevalência conforme a faixa etária foi similar ao encontrado em inquéritos realizados na Inglaterra, na Itália, no Japão, em Singapura, no Canadá, nos Estados Unidos e na Coreia do Sul^2^.

No entanto, a comparação entre os resultados de diferentes estudos deve ser feita com cautela, uma vez que a soroprevalência depende do momento da evolução da pandemia em que as pesquisas foram desenvolvidas e do teste laboratorial utilizado.

A maior soroprevalência na raça/cor preta ou parda e nos estratos sociais mais vulneráveis foi similar à encontrada em diversos estudos^20^. No Brasil, o inquérito sorológico em adultos realizado pela mesma equipe de pesquisa deste estudo no município de São Paulo^12^ e o estudo de Barros et al.^21^também evidenciaram essa disparidade.

Corroborando os estudos de Rocha et al.^22^ e Martins-Filho et al.^23^, a covid-19 foi associada a questões de vulnerabilidade socioeconômica em vez de características demográficas da população. Disparidades nos determinantes de saúde, no acesso e na qualidade dos serviços de saúde e nas condições sanitárias e de moradia aumentam o risco de doenças que podem afetar a saúde da população.

Entre aqueles que testaram positivo para a presença de anticorpos totais contra o SARS-CoV-2 e que apresentaram sintomas, os sinais clínicos mais relatados foram coriza, tosse, dor de garganta, cefaleia e febre, corroborando os relatos de outros estudos^3,5,16^.

A soroprevalência de anticorpos contra o vírus SARS-CoV-2 foi associada também ao contato com caso suspeito ou confirmado de covid-19, dado consistente com os estudos realizados no México^24^ e na China^8^. O rastreamento de contatos com o uso de testes sorológicos para detecção de indivíduos infectados pelo vírus e a avaliação da prevalência da doença na comunidade são essenciais para o conhecimento da situação epidemiológica da doença em todas as faixas etárias^25^.

Estudos mostram que o distanciamento social e o uso de medidas de proteção contra a covid-19 reduzem o contato entre as pessoas e a possibilidade de contrair a doença ao diminuir a cadeia de transmissão do vírus^26^.

Com o fechamento das escolas, foi necessária a permanência das crianças no domicílio, muitas vezes sob o cuidado de um adulto, causando aumento do absenteísmo dos pais no trabalho ou a permanência com os avós, o que pode causar aumento do risco nesse grupo^27^. Neste estudo, a proporção de escolares que permaneceram no mesmo domicílio com idosos representou quase 30,0% dos casos.

A interpretação dos achados deste estudo deve considerar algumas limitações. Dificuldades na coleta das amostras e nas entrevistas com os escolares já eram esperadas e tentaram ser compensadas no desenho amostral, aumentando-se em 50% o tamanho da amostra para se obter a precisão fixada. Os principais motivos de não resposta foram: impossibilidade de identificação do endereço ou do domicílio sorteado na base de dados, recusa dos responsáveis pelo escolar em atender a equipe de pesquisa ou do próprio escolar em querer participar do estudo, dificuldade para a equipe de pesquisa conseguir a permissão de entrada em condomínios e ausência do escolar sorteado ou do responsável pela criança no domicílio amostrado. Outros estudos da mesma natureza apontaram que falhas nas bases de dados e no uso dos instrumentos de localização, receio no atendimento à equipe e dificuldade na coleta de amostras de crianças podem ser impedimentos para realização da pesquisa de campo^28^. Este estudo manteve boa precisão das estimativas de soroprevalência de anticorpos contra o vírus SARS-CoV-2 em escolares, com erro amostral da ordem de 3%. O tamanho da amostra efetivamente coletada foi superior ao tamanho mínimo da amostra calculada. Outra limitação é que o teste laboratorial de imunoensaio de fluxo lateral que detecta anticorpos totais IgG e IgM contra o vírus SARS-CoV-2 utilizado para coleta de amostra de sangue venoso no estudo não permite determinar se a infecção é atual. Além disso, testes sorológicos podem gerar resultados falsos negativos em infecções recentes^29^.

O estudo da soroprevalência de anticorpos contra o vírus SARS-CoV-2 em crianças deve ser monitorado mesmo em locais de baixa transmissão para recomendação das medidas de proteção contra o vírus. A transmissão da doença depende do momento em que os estudos são realizados, portanto, devem ser consideradas as datas festivas, férias escolares e a diminuição das medidas de restrição^30^.

Considerando a alta capacidade de transmissão do vírus e a importância da escola para o desenvolvimento educacional, social, mental e físico das crianças e a diminuição das desigualdades sociais, econômicas e culturais da população, medidas de prevenção não farmacológicas e acompanhamento do status sorológico dos escolares são essenciais para o adequado retorno às aulas presenciais, uma vez que há maior dificuldade no seguimento de regras e higiene nessas faixas etárias, principalmente entre as populações socialmente mais vulneráveis que ainda não foram contempladas no calendário vacinal.
